# Psychosocial perspectives among cancer patients during the coronavirus disease 2019 (COVID‐19) crisis: An observational longitudinal study

**DOI:** 10.1002/cnr2.1506

**Published:** 2021-08-18

**Authors:** Ilit Turgeman, Tal Goshen‐Lago, Ithai Waldhorn, Keren Karov, Leora Groisman, Anat Reiner Benaim, Ronit Almog, Michael Halberthal, Irit Ben‐Aharon

**Affiliations:** ^1^ Division of Oncology Rambam Health Care Center Haifa Israel; ^2^ Clinical Epidemiology Unit Rambam Health Care Campus Haifa Israel; ^3^ Technion Integrated Cancer Center (TICC) Faculty of Medicine Haifa Israel; ^4^ Epidemiology Department and Biobank Rambam Health Care Campus Haifa Israel; ^5^ General Management Rambam Health Care Campus Haifa Israel

**Keywords:** clinical observations, medical oncology, psychosocial studies, quality of life

## Abstract

**Background:**

The coronavirus disease 2019 (COVID‐19) crisis and consequent changes in medical practice have engendered feelings of distress in diverse populations, potentially adversely affecting the psychological well‐being of cancer patients.

**Aim:**

The purpose of this observational longitudinal study was to evaluate psychosocial perspectives among patients with cancer on intravenous treatment during the COVID‐19 pandemic.

**Methods and results:**

The study recruited 164 cancer patients undergoing intravenous anti‐neoplastic therapy in a tertiary cancer center. Psychosocial indices were assessed at two points in time, corresponding with the beginning of the first wave of COVID‐19 pandemic in Israel (March 2020) and the time of easing of restrictions implemented to curtail spread of infection (May 2020). At Time 1 (T1), elevated COVID‐19 distress levels (score 1 and 2 on 5‐point scale) were observed in 44% of patients, and associated with pre‐existing hypertension and lung disease in multivariate analyses but no demographic or cancer related factors. At Time 2 (T2), 10% had elevated anxiety and 24% depression as indicated by Hospital Anxiety and Depression Scale (HADS‐A/D). COVID‐19 distress at T1 was related to higher levels of HADS‐A at T2 (Spearman 0.33 p < .01), but not HADS‐D. Patients with breast cancer expressed greater COVID‐19 distress compared with other cancer types (p < .01), while both HADS‐A and HADS‐D were highest for patients with GI cancer. Patient report of loneliness and decreased support from relatives were factors associated with HADS‐A (p = .03 and p < .01, respectively), while HADS‐D was not similarly related to the factors evaluated.

**Conclusion:**

Patients with cancer undergoing intravenous treatment may be vulnerable to acute adverse psychological ramifications of COVID‐19, specifically exhibiting high levels of anxiety. These appear unrelated to patient age or disease stage. Those with underlying comorbidities, breast cancer or reduced social support may be at higher risk.

## 1 INTRODUCTION

With rapid transmission across countries, variable clinical manifestation, relatively high mortality and limited therapeutic options, the novel coronavirus disease 2019 (COVID‐19) has engendered in many a state of uncertainty and panic. Unfavorable mental health has been observed among healthcare workers,^1^, ^2^ infected patients^3^ and the general population.^4^ Early on, oncological patients were presumed to be at increased risk for COVID‐19 infection, leading to modification of official treatment guidelines and preferred therapeutic modalities^5^, ^6^; these changes were based on limited evidence,^7^, ^8^ and at the potential risk of detriment to patient outcomes.^9^, ^10^ Cancer patients exhibit higher rates of anxiety and depression than those without cancer during ordinary times.^11^, ^12^ In the midst of the pandemic, the psychological impact on cancer patients has magnified, carrying both a dread of infection, as well as a concern for maintaining optimal treatment delivery — and demand for emotional assistance has soared.^5^ Many have adopted a self‐imposed lockdown. It has been shown previously that psychological distress can affect cancer progression and patient prognosis by modulating intricate bio‐behavioral processes.^13^, ^14^ Social isolation and loneliness have also been associated with mortality.^15^ On the other hand, a resilience despite the inherent distress of a cancer diagnosis has been identified in patient subgroups as a positive coping mechanism.^16^

The COVID‐19 pandemic reached Israel in February 2020, and enforcement of social distancing rules began in March 2020. On March 19, a state of national emergency was declared and a national lockdown prohibited citizens from leaving their homes unless for activities deemed essential. A gradual easing of restrictions began in early May. A second wave of COVID‐19 infection was announced during July. Located in the northern city of Haifa, Rambam Medical Center (RMC) is the tertiary referral center for 12 district hospitals, serving approximately 20% of the total nationwide population.^17^ Throughout the pandemic, cancer treatment services were maintained with minimal modifications, and patients were permitted to visit the hospital as usual.

The main objective of our study was to evaluate the psychosocial consequences of COVID‐19 on patients with cancer undergoing intravenous treatment in the oncology center during the peak of the crisis in Israel (March–May 2020). The cohort was longitudinally followed over the course of a month; first subjects completed a survey of demographics and general distress associated with COVID‐19, and ultimately, a validated assay of anxiety and depression. We evaluated indices of psychological well‐being and emotional and functional burden, considered the existence of more vulnerable subgroups of patients, and investigated a possible association between scores at different points in time.

## 2 METHODS

### 2.1 Design and procedure

The present study included patients with cancer receiving intravenous treatment administered at the ambulatory unit of the oncology center or inpatient service, within RMC. Inclusion criteria included patients of age 18 or more, with a cancer diagnosis within the past year, undergoing intravenous oncological treatment in RMC, and capable of reading and writing in the national language, Hebrew. Those with hematological malignancies were excluded, as treated in a separate department in our institution, as were patients who were not on active intravenous treatment (oral drugs, radiation or other local therapy, surgery or best supportive care). All participants signed a written informed consent. During the study period of March–May 2020, subjects completed two questionnaires for sociodemographic information as well as emotional and functional burden data. The study protocol was approved by the Institutional Ethics Committee.

### 2.2 Measures

Two questionnaires were administrated at baseline (Time 1, T1) and 1 month later (Time 2, T2) to evaluate participants' psychological state and psychosocial parameters.

#### 2.2.1 COVID‐19 distress

The T1 questionnaire available in Data S1 (Supplement 1) included demographic data, clinical variables and co‐morbidities, and a general assay of distress in relation to COVID‐19, using a scale from 1 (high distress) to 5 (low distress). Subjects were instructed to answer in relation to COVID‐19 specifically, and not a general state. A single item measure, this was meant as a general survey to determine how wary patients were of the new COVID‐19 situation.

#### 2.2.2 Anxiety and depression symptoms

At T2, after the national lockdown and period of peak of infection, and with the results of T1, it was decided to distribute a validated questionnaire to assess and quantify patient psychological indices for comparison. The Hospital Anxiety and Depression Scale (HADS) questionnaire was chosen. HADS is a 14‐item validated screening tool with two subscales for anxiety (HADS‐A) and depression (HADS‐D). For each subscale, a cut‐off HADS score of equal to or greater than 8 is considered suggestive for anxiety or depression.^18^ Designed for patients with organic diseases, HADS excludes somatic symptoms of emotional distress that may be caused by cancer, resulting in high efficacy in clinical practice,^19^ and moreover was found especially useful for screening patients in the context of COVID‐19.^20^ For the current data set, Cronbach's alpha was 0.76 (95% CI: 0.71, 0.82).

#### 2.2.3 Emotional and function burden due to the COVID‐19 pandemic

The T2 questionnaire included a series of questions designed to assess additional factors of emotional and functional burden. These were developed by the study team psychologist and social worker (available in Data S1). Parameters were analyzed as single items and a composite score was not computed.

### 2.3 Statistical analysis

Statistical analyses were performed using the R statistical software.^21^ All tests were 2‐sided with a significance level of *α* = 0.05. A multivariate rank regression model was used to analyze the association between general COVID‐19‐associated distress and demographic and co‐morbidity data, where “general COVID‐19 distress score” (ranking 1–5) was the dependent variable (Table 1). Association between “general COVID‐19 distress score” at T1 and HADS‐A and HADS‐D at T2 was assessed using Spearman's rank correlation. Univariate Fisher and Mann–Whitney tests correlated HADS‐A and D scores with key domains of emotional and functional burden.

**TABLE 1:** Rank regression with dependent variable “general anxiety score” (ranked 1–5) and variables in the following table. Adjusted model coefficients and their significance

	Estimate SD	Error	t value	p value
Female	−0.0314381	0.3141515	−0.1001	.92043
Age	0.0161412	0.0126819	1.2728	.20521
Smoker	−0.1425004	0.1177974	−1.2097	.22843
Under age 18	0.4406366	0.3543760	1.2434	.21579
Above age 70	−0.0884996	0.3219096	−0.2749	.78378
Neuro	0.8881097	1.3051239	0.6805	.49732
Lung	0.0077567	1.1849545	0.0065	.99479
Breast	−0.9636142	1.2129322	−0.7945	.42828
GU	−0.0399962	1.2540523	−0.0319	.97460
GI	−0.1179319	1.1893110	−0.0992	.92115
Gyn	0.8149090	1.4275928	0.5708	.56903
HN	−0.6308375	1.3673650	−0.4614	.64526
Sarcoma	0.3527589	1.4847099	0.2376	.81254
Melanoma	−0.7531057	1.3368464	−0.5633	.57410
Metastatic	0.0807979	0.3037710	0.2660	.79064
Heart disease	0.5851576	0.5666078	1.0327	.30351
Hypertension	−0.6947434	0.3490879	−1.9902	.04852*
Cholesterol	−0.4354029	0.5449590	−0.7990	.42566
Lung disease	−2.9187098	1.3049390	−2.2367	.02689*
Diabetes	0.0692217	0.3823836	0.1810	.85661
Asthma	0.2904382	0.8449886	0.3437	.73157

## 3 RESULTS

### 3.1 Participants

This study recruited 164 patients with cancer aged 23–90 undergoing intravenous treatment at the RHCC oncology center. Within the patient cohort, 60% had metastatic disease and 40% had loco‐regional cancer. Most common malignancies were breast (26%), lung (25%) and gastrointestinal cancer (25%), followed by genitourinary, head and neck, melanoma, gynecological and sarcoma, respectively. Treatment type included chemotherapy (74%), biological therapy (33%) and immunotherapy (25%), noting that some received more than one treatment modality. Patients were exposed to few people during the study period, suggestive of self‐isolation (p < .01). Current smoking was reported in 23% of patients, and frequent co‐morbidities included 22% hypertension and 17% diabetes mellitus. Participant characteristics are delineated in Table 2.

**TABLE 2:** Participant characteristics

Parameter	*n* (%)
Total patients	164 (100)
Gender (F)	92 (56)
*Age*	
Median	63
Range	23–90
Isolation	
Due to foreign travel	2 (1%)
Isolation due to COVID‐19 exposure	1 (0.6%)
*Living arrangement*	
Median number other individuals in household	1
Range number individuals in household	0–9
Living with people >70 years	43 (26%)
Living with people <18 years	37 (23%)
*Daily exposure to people*	
Median	0
Range	0–10
*Report of COVID‐19 related symptoms*	12 (7%)
*Comorbidities*	
Hypertension	36 (22%)
Diabetes mellitus	28 (17%)
Hyperlipidemia	15 (9%)
Heart disease	13 (8%)
Lung disease	2 (1%)
Asthma	4 (2%)
*Smoking status*	
Current smoker	38 (23%)
Former smoker	59 (36%)
Never smoker	67 (41%)

The study team requested participation from 164 patients, of these all 164 agreed and completed written consent. A full T1 questionnaire was provided by 161 patients and 99 patients went on to complete the T2 questionnaire (Table 3).

**TABLE 3:** Retention rate by cancer type

Cancer type	T1 *n* (%)	T2 *n* (%)
Total	164 (100)	99 (100)
Breast	43 (26)	34 (34)
Lung	41 (25)	25 (25)
Gastrointestinal	41 (25)	19 (19)
Genitourinary	11 (7)	4 (4)
Nervous system	8 (5)	5 (5)
Head and neck	7 (4)	4 (4)
Melanoma	6 (4)	3 (3)
Gynecological	4 (2)	3 (3)
Sarcoma	3 (2)	2 (2)

### 3.2 COVID‐19 clinical status

As of June 10, 2020, there was no documented symptomatic case of COVID‐19 infection in any study participant.

### 3.3 T1 levels of COVID‐19‐associated distress

**FIGURE 1:**
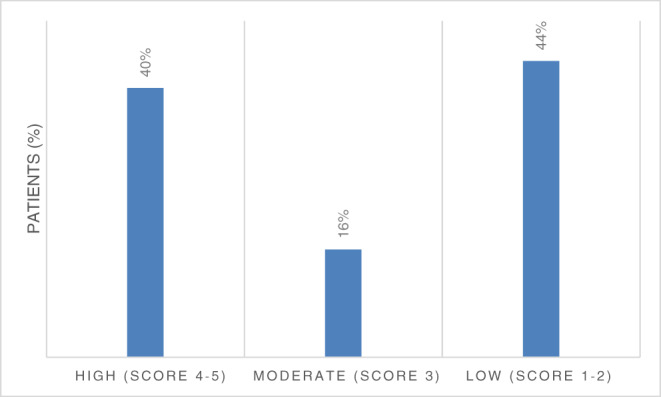
Mean COVID‐19 distress scores at T1. Of 161 study participants, the majority, or 44%, reported lower scores, signifying elevated COVID‐19 related distress levels. Lower distress was reported by 40% while only 16% had moderate scores

**FIGURE 2:**
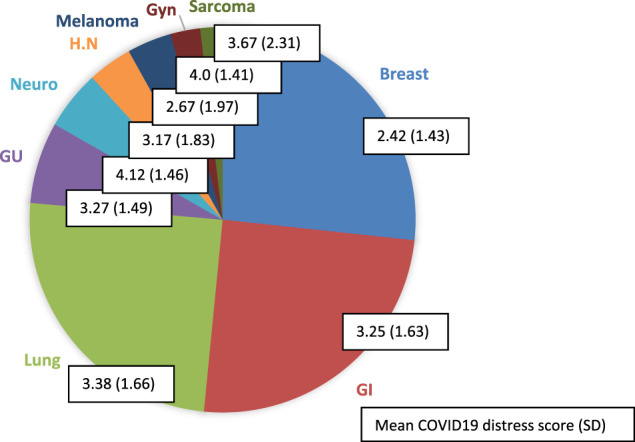
Cancer type distribution and COVID‐19 distress score. The majority of patients had breast cancer, followed by GI and lung, and the remaining had other malignancies. Following breast cancer, lowest scores, or highest distress, were reported by patients with melanoma, head and neck, GI, GU and lung cancer respectively; conversely highest scores or lowest distress was observed in subjects with neurological and gynecological malignancies.Abbreviations: GI, gastrointestinal; GU, genitourinary; GYN, gynecological; H.N, head and neck; Neuro, neurological; SD, standard deviation

The study group expressed moderate levels of distress related to COVID‐19 with mean value 3.1 (SD 1.62). However, 44% of patients had lower scores of 1 or 2, indicating greater distress (Figure 1). In multivariate analysis, demographic variables and cancer related characteristics were not correlated with distress. Association was found for patients with preexisting hypertension and lung disease, with higher distress at T1 (p = .05 SD 1.59, p = .03 SD 1.61, hypertension and lung disease, respectively). Breast cancer was associated with higher distress than other malignancies (3.3 versus 2.4 p < .01 SD 1.62), and this was maintained when adjusted for having children at home (p < .01). The limited sample size precludes inferring from data, however on a solely descriptive level, cancer types associated with higher distress scores after breast cancer included melanoma, head and neck (HN), gastrointestinal (GI), genitourinary (GU), lung cancer respectively, while lowest distress was observed in neurological and gynecological malignancies (Figure 2A,B).

### 3.4 T2 HADS score

Mean HADS‐A was 4.3 (SD 3.65), while 17 patients (10%) manifested substantial symptoms of anxiety (HADS‐A ≥ 8). Mean HADS‐D was 6.7 (3.75), while 39 patients (24%) manifested substantial symptoms of depression (HADS‐D ≥ 8).

**FIGURE 3:**
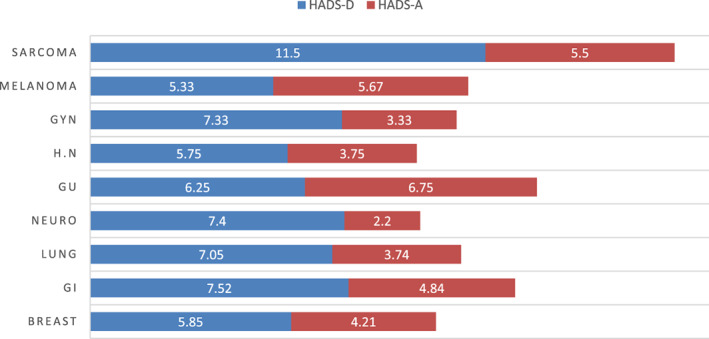
Hospital anxiety and depression scale (HADS) scores at T2 by cancer type. Of groups with over 15 subjects, HADS‐A was highest among GI and breast cancer patients, and HADS‐D in those with GI cancer, however HADS‐D was lowest among patients with breast cancer. Despite small sample size, a largely abnormal mean depression score of over 11 was observed only in patients with sarcoma

Of groups with over 15 subjects, HADS‐A was highest among breast cancer patients, and HADS‐D in those with GI cancer, however HADS‐D was lowest among patients with breast cancer (Figure 3 and Table 4). Despite small sample size, a largely abnormal mean depression score of over 11 was observed only in patients with sarcoma. Anxiety (HADS≥8) was not associated with gender, age, smoking, co‐morbidities, living arrangement, financial situation, communication with family, or news consumption. Participants who reported decreased support from relatives had higher HADS‐A scores, (p < .01). Furthermore, positive association was found between loneliness and HADS‐A (p < .01). Finally, subjects with lower HADS‐A were more likely to express optimism for the future regarding COVID‐19 (p = .03). Associations of demography, cancer or emotional and functional burden parameters were not found for HADS‐D. Parameters of HADS scores by cancer type can be found in Data S1 (Supplement 2).

**TABLE 4:** Mean and median HADS‐A and HADS‐D scores by cancer type

HADS	Cancer type	*n*	Mean score	Median score	SD
HADS‐A	Breast	34	4.21	3	3.67
	GI	25	4.84	3	4.16
	Lung	19	3.74	4	3.49
	Neuro	5	2.2	1	2.95
	GU	4	6.75	8	4.99
	HN	4	3.75	3.5	1.71
	Gyn	3	3.33	3	2.52
	Melanoma	3	5.67	5	3.06
	Sarcoma	2	5.5	5.5	2.12
HADS‐D	Breast	34	5.85	5	2.97
	GI	25	7.52	7	4.33
	Lung	19	7.05	6	4.33
	Neuro	5	7.4	8	1.82
	GU	4	6.25	7.5	4.5
	H.N	4	5.75	6	3.77
	Gyn	3	7.33	11	6.35
	Melanoma	3	5.33	6	2.08
	Sarcoma	2	11.5	11.5	2.12

### 3.5 Association: T1‐T2

**FIGURE 4:**
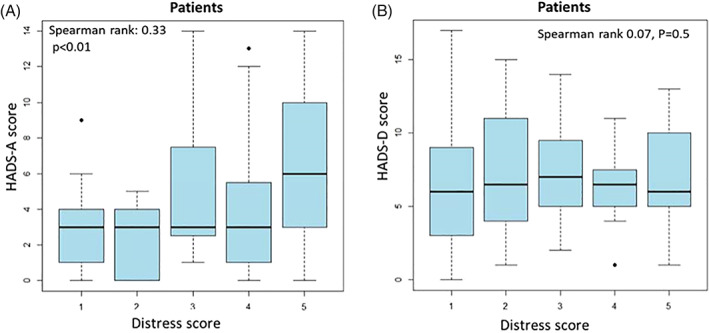
Spearman rank correlation between COVID‐19 distress at T1 and HADS scores at T2. Patients with higher initial COVID‐19 distress scores at T1, later manifested greater anxiety at T2 (A). Similar results were not demonstrated for depression; at T2, depression levels centered homogeneously around 6 and were not associated with COVID‐19 distress at T1 (B)

Significant association over time is observed for anxiety; patients with higher degree of COVID‐19 distress at T1 demonstrated higher HADS‐A scores at T2 (Spearman rank 0.33 p < .01) (Figure 4A). Regarding depression, similar positive association was not observed (Spearman rank 0.07, p = .5). While T1 scores varied between study participants (40% low; 16% moderate; 44% high), HADS‐D scores were centered homogeneously around 6, suggesting less variation in depressive symptoms between individual patients (Figure 4B).

## 4 DISCUSSION

The COVID‐19 crisis has posed a new and imminent threat to the continuity of care in cancer patients, possibly compounding any existing distress related to their cancer and future outlook. This has been previously assessed in different populations, points in time and locations worldwide. Cross‐sectional studies in China showed high anxiety and depression among the general population,^22^ negative mental health outcomes in frontline healthcare workers,^1^ and depressive symptoms were even observed among school‐age children.^23^ As the pandemic spread to Europe, similar studies exposed mental health issues in Spanish populations,^24^ healthcare workers in Italy,^2^ and more. A review of 62 studies from 17 countries found high mental burden among medical staff and general public, however distress was most evident among patients.^25^ Studies generally delineate psychological indices from a snapshot in time, rather than a longitudinal follow‐up more likely to mitigate the chance of an acute surge in distress that abated over time.

The present study aimed to longitudinally evaluate psychosocial perspectives of patients with cancer undergoing intravenous treatment during the time of peak COVID‐19 infection, and found initially elevated COVID‐19 distress levels that predicted patient anxiety after 1 month, as well as loneliness and feelings of decreased social support. Nevertheless, similar correlations were not found for depression. Compared to the general population, depression is known to be relatively more common among cancer patients, especially those with metastatic disease. In the present study, depression levels were unequivocally higher than anxiety but homogenous and not correlated with COVID‐19 distress; anxiety levels, however, were heterogenous and significantly linked to COVID‐19 distress – thereby highlighting this distinct aspect of psychological well‐being as a potentially representative aspect of the experience of patients with cancer in the COVID‐19 era. As above, former studies evaluating non‐cancer patients did demonstrate COVID‐19‐associated depression. Taken together, these data suggest that anxiety may be an effective indicator of the mental state of cancer patients in relation to COVID‐19, while depression a more ingrained and enduring trait that is less affected by acute stress. To illustrate this, patients with breast cancer had high COVID‐distress and HADS‐A scores, but lower HADS‐D.

This study identifies subgroups of patients who may be at greater risk for emotional distress and thereby benefit from early psychological support. The susceptibility of patients with underlying conditions or even poor perceived health is widely corroborated by other studies^4^, ^25^— and in line with their higher risk to contract severe COVID‐19 illness.^26^ Furthermore, pervasiveness of distress in breast cancer patients compared to those with other malignancies is well researched and often influenced by social context.^27^ Female gender, news consumption and having symptoms suggestive of COVID‐19 were previously identified as risk factors,^28^ but not shown here. Patients of younger age and with localized disease have been shown to manifest resilience to psychological distress in spite of a cancer diagnosis.^16^ Here, age and stage did not affect distress, suggesting that general parameters of prognosis do not allay COVID‐stress response. The lack of difference between cancer type and stage may be due to patient receipt of intravenous treatment, which may be a factor in determining vulnerability. This analysis evaluated patients receiving intravenous treatment, which may define a group of patients with cancer who are more aware of reductions in immune system function and possible susceptibility to COVID‐19 infection and complications, as opposed to those treated with surgery, receiving radiotherapy or other local treatment, oral medication at home or none at all. Finally, social support is considered an important contributor to improving well‐being and reducing distress in cancer patients both in ordinary and extraordinary times.^16^

In international databases, a steep decline in oncology‐related procedures and visits has been observed.^28^, ^29^ It has been suggested that the dread of “unnecessary” hospital visits, especially at the interface with the emergency department, may make cancer patients reluctant to seek medical help in acute situations. This conjecture warrants further investigation in larger controlled studies. The possibility that patients avoid hospital visits could have detrimental consequences on outcome, with potential delayed diagnoses of treatment‐related adverse events, oncological emergencies or disease progression.

This study has several limitations. Firstly, a small sample size taken from a single institution was utilized. Patients chose to participate perhaps affecting degree of distress or willingness to partake in the study. The study cohort included mostly patients with metastatic disease, which may have influenced the results as a subpopulation more at risk and more distressed by COVID‐19. These reflect the patient distribution in the RMC oncology daycare. Importantly, COVID‐19 distress and HADs were not evaluated at both time points, rather one was compared to the other, limiting the strength of the data. At T1, a general survey was conducted to assess the degree of COVID‐19 distress, and as the epidemic progressed and the lockdown continued, the need arose for a validated quantification of patient distress, or the HADs survey. This does not represent change over time but rather a longitudinal comparison of separate factors, or initial COVID‐distress and eventual psychological state. In other words, those who were weary of COVID‐19 also eventually demonstrated high anxiety levels, which was not true for depression, thus isolating anxiety as a unique COVID‐19 related issue and providing further distinction between cancer‐anxiety and COVID‐19‐anxiety. It can be assumed that actual levels may in fact be higher as patients who did not arrive for treatment due to self‐isolation were not represented. Large interventional controlled trials are needed to support findings of patient distress and determine ramifications on patient treatment or outcome.

While in some parts of the world the pandemic is abating, in other parts, peak levels are being experienced, and in still others, harbingers of additional waves are emerging — regardless, oncology teams should anticipate emotional distress among patients and prepare in advance. Availability of accurate information and the use of precautionary measures as hand‐washing and wearing a mask, have been shown to correlate with lower COVID‐19‐related anxiety and depression.^29^ Evidence‐based clear guidelines and detailed treatment plans should be conveyed and implemented. Active interventions should be pursued, such as psychological counseling, interactive virtual meetings, encouragement of maintaining healthy diet and exercise.^5^ Meaningful guidance and multidisciplinary support should aim to reduce feelings of hopelessness and lack of control. In conclusion, sufficient attention should be reserved for the psychological care of cancer patients concerning the current pandemic, with the aim of alleviating anxiety especially at vulnerable points in time, in order to minimize risk without compromising care.

## CONFLICT OF INTEREST

Authors have no conflicts of interests to disclose.

## AUTHOR CONTRIBUTIONS

All authors had full access to the data in the study and take responsibility for the integrity of the data and the accuracy of the data analysis. *Conceptualization*, I.T., T.G.L., K.K, L.G., I.B.A.; *Methodology*, I.T., T.G.L., I.B.A.; *Investigation*, I.T., T.G.L., I.B.A.; *Formal analysis*, I.T., T.G.L., I.W., A.R.B., R.A., I.B.A.; *Resources*, I.T., T.G.L.*; Writing‐Original draft*, I.T., T.G.L., I.W., I.B.A.; *Writing‐Review & Editing*, I.T., T.G.L., I.W., M.H., I.B.A.; *Visualization*, I.T., T.G.L., I.W., A.R.B., R.A., I.B.A.; *Data Curation*, I.T., T.G.L., I.B.A.; *Validation*, I.T., T.G.L., I.B.A.; *Project Administration*, M.H., I.B.A.

## ETHICAL STATEMENT

The study protocol was approved by the Institutional Ethics Committee (RHCC; RMB 0209–20). Patients provided written informed consent to participate in the study.

## Supporting information


**Data S1.** Supplementary material

## Data Availability

Extra data can be accessed by emailing the corresponding author.

## References

[1] Lai J , Ma S , Wang Y , et al. Factors associated with mental health outcomes among health care workers exposed to coronavirus disease 2019. JAMA Netw Open. 2020;3(3):e203976. 32202646 10.1001/jamanetworkopen.2020.3976 PMC7090843

[2] Rossi R , Socci V , Pacitti F , et al. Mental health outcomes among frontline and second‐line health care workers during the coronavirus disease 2019 (COVID‐19) pandemic in Italy. JAMA Netw Open. 2020;3(5):e2010185. 32463467 10.1001/jamanetworkopen.2020.10185 PMC7256664

[3] Bo HX , Li W , Yang Y , et al. Posttraumatic stress symptoms and attitude toward crisis mental health services among clinically stable patients with COVID‐19 in China. Psychol Med. 2020;51(6):1052‐1053. 32216863 10.1017/S0033291720000999 PMC7200846

[4] Wang C , Pan R , Wan X , et al. Immediate psychological responses and associated factors during the initial stage of the 2019 coronavirus disease (COVID‐19) epidemic among the general population in China. Int J Environ Res Public Health. 2020;17(5):1729. 10.3390/ijerph17051729 PMC7084952 32155789

[5] van de Haar J , Hoes LR , Coles CE , et al. Caring for patients with cancer in the COVID‐19 era. Nat Med. 2020;26:665‐671. 32405058 10.1038/s41591-020-0874-8

[6] Bartlett DL , Howe JR , Chang G , et al. Management of cancer surgery cases during the COVID‐19 pandemic: considerations. Ann Surg Oncol. 2020;27:1717‐1720. 32270420 10.1245/s10434-020-08461-2 PMC7141488

[7] Uzzo RG , Kutikov A , Geynisman DM . Coronavirus disease 2019 (COVID‐19): Cancer care during the pandemic. UpToDate, Post, TW. Waltham, MA: UpToDate; 2020 .

[8] Cannistra SA , Haffty BG , Ballman K . Challenges faced by medical journals during the COVID‐19 pandemic [published online ahead of print, 2020 April 8]. J Clin Oncol. 2020;2020:JCO2000858. 10.1200/JCO.20.00858 32267782

[9] Wang H , Zhang L . Risk of COVID‐19 for patients with cancer. Lancet Oncol. 2020;21(4):e181. 32142621 10.1016/S1470-2045(20)30149-2 PMC7129735

[10] Nagar H , Formenti SC . Cancer and COVID‐19 — potentially deleterious effects of delaying radiotherapy. Nat Rev Clin Oncol. 2020;17:332‐334. 32341524 10.1038/s41571-020-0375-1 PMC7184542

[11] Krebber AM , Buffart LM , Kleijn G , et al. Prevalence of depression in cancer patients: a meta‐analysis of diagnostic interviews and self‐report instruments. Psychooncology. 2014;23(2):121‐130. 24105788 10.1002/pon.3409 PMC4282549

[12] Spencer R , Nilsson M , Wright A , Pirl W , Prigerson H . Anxiety disorders in advanced cancer patients: correlates and predictors of end‐of‐life outcomes. Cancer. 2010;116(7):1810‐1819. 20187099 10.1002/cncr.24954 PMC2846995

[13] Reis JC , Antoni MH , Travado L . Emotional distress, brain functioning, and biobehavioral processes in cancer patients: a neuroimaging review and future directions. CNS Spectr. 2020;25(1):79‐100. 31010446 10.1017/S1092852918001621

[14] Bortolato B , Hyphantis TN , Valpione S . Depression in cancer: the many biobehavioral pathways driving tumor progression. Cancer Treat Rev. 2017;52:58‐70. 27894012 10.1016/j.ctrv.2016.11.004

[15] Holt‐Lunstad J , Smith TB , Baker M , Harris T , Stephenson D . Loneliness and social isolation as risk factors for mortality: a meta‐analytic review. Perspect Psychol Sci. 2015;10(2):227‐237. 25910392 10.1177/1745691614568352

[16] Seiler A , Jenewein J . Resilience in cancer patients. Front Psych. 2019;10:208. 10.3389/fpsyt.2019.00208 PMC6460045 31024362

[17] https://www.rambam.org.il/en/

[18] Zigmond AS , Snaith RP . The hospital anxiety and depression scale. Acta Psychiatr Scand. 1983;67(6):361‐370. 6880820 10.1111/j.1600-0447.1983.tb09716.x

[19] Annunziata MA , Muzzatti B , Bidoli E . Hospital anxiety and depression scale (HADS) accuracy in cancer patients. Support Care Cancer. 2020;28(8):3921‐3926. 31858249 10.1007/s00520-019-05244-8

[20] Curigliano G , Cardoso MJ , Poortmans P , et al. Recommendations for triage, prioritization and treatment of breast cancer patients during the COVID‐19 pandemic [published online ahead of print, 2020 April 16]. Breast. 2020;52:8‐1. 32334323 10.1016/j.breast.2020.04.006 PMC7162626

[21] Core Team R . R: A Language and Environment for Statistical Computing. Vienna, Austria: R Foundation for Statistical Computing; 2020 https://www.R-project.org/

[22] Li J , Yang Z , Qiu H , et al. Anxiety and depression among general population in China at the peak of the COVID‐19 epidemic. World Psychiatry. 2020;19(2):249‐250. 32394560 10.1002/wps.20758 PMC7214959

[23] Xie X , Xue Q , Zhou Y , et al. Mental health status among children in home confinement during the coronavirus disease 2019 outbreak in Hubei Province. China JAMA Pediatr. 2020;174(9):898–900. 32329784 10.1001/jamapediatrics.2020.1619 PMC7182958

[24] Ozamiz‐Etxebarria N , Dosil‐Santamaria M , Picaza‐Gorrochategui M , Idoiaga‐Mondragon N . Stress, anxiety, and depression levels in the initial stage of the COVID‐19 outbreak in a population sample in the northern Spain. Cad Saude Publica. 2020;36(4):e00054020. 32374806 10.1590/0102-311X00054020

[25] Luo M , Guo L , Mingzhou Y , Wang H . The psychological and mental impact of coronavirus disease 2019 (COVID‐19) on medical staff and general public – a systematic review and meta‐analysis. Psychiatry Res. 2020;291:113190. 32563745 10.1016/j.psychres.2020.113190 PMC7276119

[26] Dong XC , Li JM , Bai JY , et al. Epidemiological characteristics of confirmed COVID‐19 cases in Tianjin. Zhonghua Liu Xing Bing Xue Za Zhi. 2020;41:638‐642. 32164400 10.3760/cma.j.cn112338-20200221-00146

[27] Niedzwiedz CL , Knifton L , Robb KA , Katikireddi SV , Smith DJ . Depression and anxiety among people living with and beyond cancer: a growing clinical and research priority. BMC Cancer. 2019;19:943. 31604468 10.1186/s12885-019-6181-4 PMC6788022

[28] IQVIA Institute for Human Data Science . Shifts in healthcare demand, delivery and care during the COVID‐19 era . 29 April 2020.

[29] MDedge . Cancer screening, monitoring down during pandemic. 4 May 2020.

