# Supplementary material for: Psychosocial perspectives among cancer patients during the coronavirus disease 2019 (COVID‐19) crisis: An observational longitudinal study
Source: Cancer Rep (Hoboken). 2021 Aug 18;5(5):e1506. doi: 10.1002/cnr2.1506 (PMC8420321; doi:10.1002/cnr2.1506)
Supplement: Supplementary file 1 — Data S1. Supplementary material [file CNR2-5-e1506-s001.docx]

**Supplementary material**

Supplement 1: Subject questionnaires

**T1 questionnaire: COVID-19 exposure and distress**

Subject number:____ Age:__

Gender: m/f Occupation:__

City of residence:__

Comorbidity:__

Smoking status: daily/ past (5y)/ past (>5)/ never

Quarantine: never/ due to foreign travel/ due to exposure/ self-proclaimed Date:_

Symptoms of COVID-19 currently (fever, cough, throat pain, loss of taste/smell):__

Distress associated specifically with COVID-19 (1-very distressed, 5-not distressed at all): __

Number of people exposed to daily, non-family: __

Number of family members in household: __ Above age 70: __ Below age 18: __

**T2 questionnaire: HADS score and emotional coping parameters**

Please answer the following questions according to how you feel today, regarding the Corona crisis

1. Did the corona crisis cause a decrease in communication between you and your family members living with you at home?

A. There was no decrease in communication

B. There has been a decline in communication

C. Communication has diminished and at times I feel disconnected from them

D. The communication has diminished greatly and I feel very lonely

2. To what extent are you assisted by family members or other significant relatives during this period?

A. Equally aided

B. Helped less than before

C. Helped more than ever

3. How many hours a day do you consume news (internet, newspaper, TV)?

A. Not at all

B. Up to two hours a day

C. Between two and five hours a day

D. Five hours or more

4. To what extent do you feel lonely during this period?

A. Not at all

B. Slightly

C. Moderately

D. very much

5. To what extent has there been a change in your financial situation during this period?

A. no change

B. Slightly

C. Moderately

D. very much

6. To what extent are you concerned about a change in your financial situation?

A. Not at all

B. Slightly

C. Moderately

D. very much

7. What will you save regarding the future due to the corona crisis?

A. optimistic

B. pessimistic

C. I do not care

Supplement 2: Hospital Anxiety and Depression Scale (HADS) results by cancer type

Score 0-3 for question (0-least; 3-most)

Score 0-7 = Normal; 8-10 = Borderline abnormal; 11-21 = Abnormal

| Measure | cancer | n | mean | median | sd |
| --- | --- | --- | --- | --- | --- |
| I feel tense or 'wound up | Neuro | 5 | 0.2 | 0 | 0.45 |
|  | Lung | 19 | 0.47 | 0 | 0.7 |
|  | Breast | 34 | 0.85 | 0.5 | 1.02 |
|  | GU | 4 | 1.25 | 1.5 | 0.96 |
|  | GI | 25 | 0.6 | 0 | 0.82 |
|  | Gyn | 3 | 0.33 | 0 | 0.58 |
|  | H.N | 4 | 0.25 | 0 | 0.5 |
|  | Sarcoma | 2 | 1 | 1 | 1.41 |
|  | Melanoma | 3 | 1 | 1 | 1 |
| I still enjoy the things I used to enjoy | Neuro | 5 | 2.4 | 3 | 0.89 |
|  | Lung | 19 | 1.58 | 2 | 1.22 |
|  | Breast | 34 | 1.26 | 1 | 1.19 |
|  | GU | 4 | 1 | 1 | 0.82 |
|  | GI | 25 | 1.32 | 1 | 1.14 |
|  | Gyn | 3 | 1.33 | 1 | 1.53 |
|  | H.N | 4 | 0.75 | 0.5 | 0.96 |
|  | Sarcoma | 2 | 2.5 | 2.5 | 0.71 |
|  | Melanoma | 3 | 0.67 | 0 | 1.15 |
| I get a sort of frightened feeling as if something awful is about to happen | Neuro | 5 | 0.2 | 0 | 0.45 |
|  | Lung | 19 | 0.32 | 0 | 0.58 |
|  | Breast | 34 | 0.47 | 0 | 0.75 |
|  | GU | 4 | 1 | 1 | 0.82 |
|  | GI | 25 | 0.56 | 0 | 0.92 |
|  | Gyn | 3 | 0 | 0 | 0 |
|  | H.N | 4 | 0.5 | 0 | 1 |
|  | Sarcoma | 2 | 0 | 0 | 0 |
|  | Melanoma | 3 | 0.67 | 0 | 1.15 |
| I can laugh and see the funny side of things: | Neuro | 5 | 2 | 3 | 1.41 |
|  | Lung | 19 | 1.53 | 1 | 1.22 |
|  | Breast | 34 | 1.03 | 1 | 1.06 |
|  | GU | 4 | 1.5 | 1.5 | 1.29 |
|  | GI | 24 | 1.42 | 1 | 1.18 |
|  | Gyn | 3 | 1 | 1 | 1 |
|  | H.N | 4 | 1 | 0.5 | 1.41 |
|  | Sarcoma | 2 | 0.5 | 0.5 | 0.71 |
|  | Melanoma | 3 | 0.33 | 0 | 0.58 |
| Worrying thoughts go through my mind: | Neuro | 5 | 0.4 | 0 | 0.89 |
|  | Lung | 19 | 0.68 | 0 | 0.82 |
|  | Breast | 34 | 0.85 | 1 | 0.89 |
|  | GU | 4 | 1.5 | 1.5 | 1.29 |
|  | GI | 24 | 0.96 | 1 | 1.04 |
|  | Gyn | 3 | 1 | 1 | 0 |
|  | H.N | 4 | 0.75 | 1 | 0.5 |
|  | Sarcoma | 2 | 0.5 | 0.5 | 0.71 |
|  | Melanoma | 3 | 1.67 | 2 | 0.58 |
| I feel cheerful: | Neuro | 4 | 0.75 | 0 | 1.5 |
|  | Lung | 19 | 1.63 | 1 | 1.12 |
|  | Breast | 34 | 1.18 | 1 | 0.97 |
|  | GU | 4 | 0.75 | 0.5 | 0.96 |
|  | GI | 24 | 1.54 | 2 | 1.06 |
|  | Gyn | 3 | 1.33 | 1 | 1.53 |
|  | H.N | 4 | 1.25 | 1 | 0.5 |
|  | Sarcoma | 2 | 2 | 2 | 1.41 |
|  | Melanoma | 3 | 1.33 | 1 | 0.58 |
| I can sit at ease and feel relaxed | Neuro | 5 | 0.6 | 0 | 0.89 |
|  | Lung | 19 | 1.11 | 1 | 1.24 |
|  | Breast | 33 | 0.79 | 1 | 0.86 |
|  | GU | 4 | 0.75 | 0.5 | 0.96 |
|  | GI | 25 | 1.28 | 1 | 1.06 |
|  | Gyn | 3 | 0.67 | 0 | 1.15 |
|  | H.N | 4 | 1.25 | 1 | 1.26 |
|  | Sarcoma | 2 | 1.5 | 1.5 | 0.71 |
|  | Melanoma | 3 | 1 | 1 | 1 |
| I feel as if I am slowed down: | Neuro | 5 | 1.8 | 2 | 1.3 |
|  | Lung | 19 | 0.53 | 0 | 0.84 |
|  | Breast | 34 | 0.68 | 1 | 0.64 |
|  | GU | 4 | 1.25 | 1.5 | 0.96 |
|  | GI | 25 | 1 | 1 | 1.08 |
|  | Gyn | 3 | 0.67 | 0 | 1.15 |
|  | H.N | 4 | 1 | 1 | 1.15 |
|  | Sarcoma | 2 | 2.5 | 2.5 | 0.71 |
|  | Melanoma | 3 | 1.33 | 1 | 1.53 |
| I get a sort of frightened feeling like 'butterflies' in the stomach: | Neuro | 5 | 0.8 | 0 | 1.3 |
|  | Lung | 19 | 0.32 | 0 | 0.67 |
|  | Breast | 34 | 0.47 | 0 | 0.66 |
|  | GU | 4 | 0.75 | 0.5 | 0.96 |
|  | GI | 24 | 0.5 | 0 | 0.83 |
|  | Gyn | 3 | 0.33 | 0 | 0.58 |
|  | H.N | 4 | 0.5 | 0.5 | 0.58 |
|  | Sarcoma | 2 | 2 | 2 | 1.41 |
|  | Melanoma | 3 | 0.67 | 1 | 0.58 |
| I have lost interest in my appearance: | Neuro | 5 | 0.2 | 0 | 0.45 |
|  | Lung | 19 | 0.37 | 0 | 0.9 |
|  | Breast | 33 | 0.42 | 0 | 0.75 |
|  | GU | 4 | 0.25 | 0 | 0.5 |
|  | GI | 25 | 0.68 | 0 | 0.99 |
|  | Gyn | 3 | 1 | 1 | 1 |
|  | H.N | 3 | 0.33 | 0 | 0.58 |
|  | Sarcoma | 2 | 0.5 | 0.5 | 0.71 |
|  | Melanoma | 3 | 0.33 | 0 | 0.58 |
| I feel restless as I have to be on the move: | Neuro | 5 | 0 | 0 | 0 |
|  | Lung | 19 | 0.68 | 0 | 0.82 |
|  | Breast | 34 | 0.47 | 0 | 0.75 |
|  | GU | 4 | 0.75 | 0.5 | 0.96 |
|  | GI | 25 | 0.72 | 1 | 0.79 |
|  | Gyn | 3 | 0.33 | 0 | 0.58 |
|  | H.N | 4 | 0.25 | 0 | 0.5 |
|  | Sarcoma | 2 | 0 | 0 | 0 |
|  | Melanoma | 3 | 0.67 | 1 | 0.58 |
| I look forward with enjoyment to things: | Neuro | 5 | 0.2 | 0 | 0.45 |
|  | Lung | 19 | 0.95 | 1 | 0.97 |
|  | Breast | 32 | 0.81 | 0.5 | 1.03 |
|  | GU | 4 | 0.75 | 1 | 0.5 |
|  | GI | 24 | 1 | 1 | 1.06 |
|  | Gyn | 3 | 1 | 1 | 1 |
|  | H.N | 4 | 1.25 | 1.5 | 0.96 |
|  | Sarcoma | 2 | 2.5 | 2.5 | 0.71 |
|  | Melanoma | 3 | 1.33 | 1 | 0.58 |
| I get sudden feelings of panic: | Neuro | 5 | 0 | 0 | 0 |
|  | Lung | 19 | 0.16 | 0 | 0.5 |
|  | Breast | 34 | 0.32 | 0 | 0.64 |
|  | GU | 4 | 0.75 | 1 | 0.5 |
|  | GI | 25 | 0.28 | 0 | 0.54 |
|  | Gyn | 3 | 0.67 | 0 | 1.15 |
|  | H.N | 4 | 0.25 | 0 | 0.5 |
|  | Sarcoma | 2 | 0.5 | 0.5 | 0.71 |
|  | Melanoma | 3 | 0 | 0 | 0 |
| I can enjoy a good book or radio or TV program | Neuro | 5 | 0.2 | 0 | 0.45 |
|  | Lung | 19 | 0.47 | 0 | 0.9 |
|  | Breast | 34 | 0.53 | 0 | 0.79 |
|  | GU | 4 | 0.75 | 0.5 | 0.96 |
|  | GI | 25 | 0.72 | 0 | 0.94 |
|  | Gyn | 3 | 1 | 1 | 1 |
|  | H.N | 4 | 0.25 | 0 | 0.5 |
|  | Sarcoma | 2 | 1 | 1 | 0 |
|  | Melanoma | 3 | 0 | 0 | 0 |
